# Optimizing Input Selection for Cardiac Model Training and Inference: An Efficient 3D Convolutional Neural Networks-Based Approach to Automate Coronary Angiogram Video Selection

**DOI:** 10.1016/j.mcpdig.2025.100195

**Published:** 2025-01-21

**Authors:** Shih-Sheng Chang, Behrouz Rostami, Gerardo LoRusso, Chia-Hao Liu, Mohamad Alkhouli

**Affiliations:** a Department of Cardiovascular Medicine, Mayo Clinic, Rochester, MN; b Division of Cardiology, Department of Internal Medicine, China Medical University Hospital, Taichung, Taiwan; c Artificial Intelligence Center, China Medical University Hospital, Taichung, Taiwan; d School of Medicine, College of Medicine, China Medical University, Taichung, Taiwan; e Department of Clinical Sciences and Community Health, Cardiovascular Section, University of Milan, Milan, Italy

## Abstract

**Objective:**

To develop an efficient and automated method for selecting appropriate coronary angiography videos for training deep learning models, thereby improving the accuracy and efficiency of medical image analysis.

**Patients and Methods:**

We developed deep learning models using 232 coronary angiographic studies from the Mayo Clinic. We utilized 2 state-of-the-art convolutional neural networks (CNN: ResNet and X3D) to identify low-quality angiograms through binary classification (satisfactory/unsatisfactory). Ground truth for the quality of the input angiogram was determined by 2 experienced cardiologists. We validated the developed model in an independent dataset of 3208 procedures from 3 Mayo sites.

**Results:**

The 3D-CNN models outperformed their 2D counterparts, with the X3D-L model achieving superior performance across all metrics (AUC 0.98, accuracy 0.96, precision 0.87, and F1 score 0.92). Compared with 3D models, 2D architectures are smaller and less computationally complex. Despite having a 3D architecture, the X3D-L model had lower computational demand (19.34 Giga Multiply Accumulate Operation) and parameter count (5.34 M) than 2D models. When validating models on the independent dataset, slight decreases in all metrics were observed, but AUC and accuracy remained robust (0.95 and 0.92, respectively, for the X3D-L model).

**Conclusion:**

We developed a rapid and effective method for automating the selection of coronary angiogram video clips using 3D-CNNs, potentially improving model accuracy and efficiency in clinical applications. The X3D-L model reports a balanced trade-off between computational efficiency and complexity, making it suitable for real-life clinical applications.

## Graphical abstract

**Figure:**
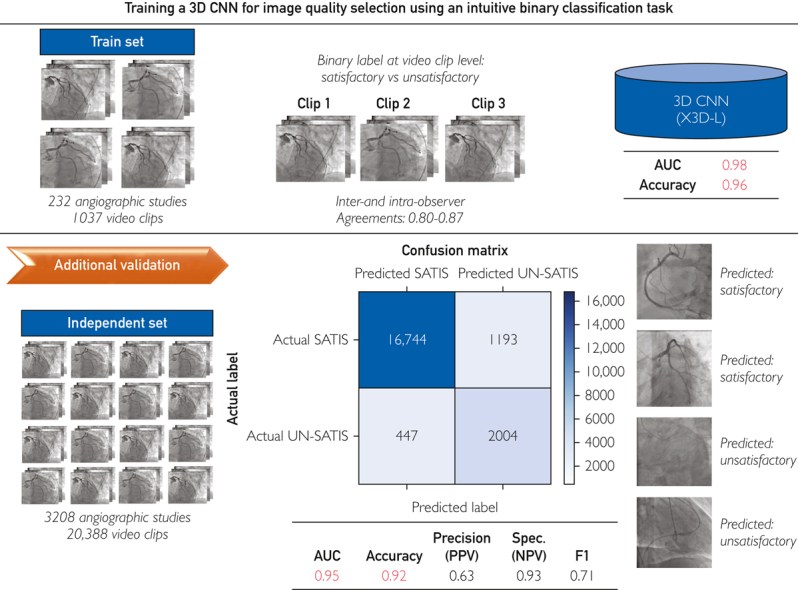

As machine learning (ML) becomes more integrated into cardiology, there is an increasing trend of utilizing invasive X-ray coronary angiograms for ML-driven analyses. This approach allows for evaluating left ventricular systolic function,^1^ coronary artery stenosis,^2^ and myocardial ischemia.^3^^,^^4^ Coronary angiogram videos are an essential data source for ML models, with their performance heavily dependent on the careful selection of appropriate Digital Imaging and Communications in Medicine images. The process is challenging because of variability in image acquisition, background noise, and the frequent irrelevant or nondiagnostic images (eg, short cine to verify puncture site or catheter location). These issues hinder the performance of the best ML models. Poor catheter engagement, implanted devices, or suboptimal contrast filling further degrade image quality and model accuracy.^5^^,^^6^ This highlights the need for efficient techniques to select the optimal images for model training and testing.

Automated methods like keyframe selection and object detection have improved selection efficiency^2^^,^^7^ but are often designed for still images, making them less suitable for video angiographic clips. These methods require large datasets and extensive annotations. Therefore, we developed a workflow (Graphic Abstract) leveraging advanced convolutional neural networks (CNN), such as 2D (ResNet 2D) and 3D (ResNet 3D and X3D) architectures. ResNet 2D models focus on spatial features in images, whereas ResNet 3D models are designed to capture temporal information.^8^^,^^9^ The X3D model further improved processing efficiency and reported success in predicting left ventricular function.^1^ This automated tool could streamline preprocessing, enhance downstream model accuracy, and ensure high-quality input, eliminating the need for manual selection.

## Methods

### Data Selection

The utilized dataset of cardiac catheterization imaging was meticulously curated by enlisting adult patients who underwent coronary angiography at Mayo Clinic locations in Minnesota, Florida, and Arizona from January 1, 2010, to December 31, 2021. An institutional review board approval was obtained. Patients who did not consent to use their medical records for research were excluded. A random sampling methodology was employed to select 232 cardiac catheterization examinations, which included 1037 videos, to develop our quality selection model. Subsequently, a larger independent coronary angiogram dataset, consisting of 3208 cardiac catheterization examinations and 20,388 videos, was used to validate the model.

### Annotation Criteria

We annotated video clips in each angiographic study instead of making binary selections to avoid omitting potentially useful clips for model training. Two board-certified cardiologists reviewed the clips independently. The following criteria were utilized to classify video clips as unsatisfactory: (1) noncoronary angiograms, such as angiograms of the aorta, left ventricle, or other structures; (2) angiography images in which the coronary arteries are obscured due to improper catheter engagement, inadequate contrast filling, or partial recordings during percutaneous coronary interventions; (3) angiography images of graft vessels in patients who have undergone coronary artery bypass operation; (4) angiograms with foreign objects, like pacemakers or spinal implants, obstruct coronary arteries, complicating model discernment. We closely monitored each image to exclude only those angiograms where foreign objects considerably hindered visualization of most coronary arteries. This ensures quality assurance and model generalizability, avoiding unnecessary video discards.

### Model Training

To prevent data leakage, we divided the angiogram images into training, validation, and test sets on the basis of unique patient IDs, with proportions of 70%, 15%, and 15%, respectively. In this study, both 2D and 3D CNN architectures (ResNet-18, ResNet-152, and X3D) were employed to evaluate the quality of the angiograms.^10^, ^11^, ^12^ Frames were extracted from angiogram videos to optimize analysis, and the models were trained using standard machine-learning techniques with data augmentation to improve generalizability. Detailed technical settings are provided in the Supplemental Material.

### Model Evaluation

The efficacy of our models in classifying angiogram images as satisfactory (negative class) or unsatisfactory (positive class) was assessed using the following metrics: the area under the receiver operating characteristic curve (AUC), accuracy, precision, sensitivity, specificity, and F1 score.^13^^,^^14^ The AUC reflects the model’s discriminative ability by plotting sensitivity versus 1-specificity, with higher values indicating superior performance. Accuracy, representing the proportion of true results (both true positives and true negatives) in the total number of cases examined. Precision, also known as positive predictive value, is the ratio of true positive predictions to the total number of positive predictions. The F1 score is a harmonic mean of precision and recall (sensitivity). The F1 score balances the trade-off between precision and recall, which is particularly relevant in the face of class imbalance.

To assess the model’s complexity and efficiency, we used the ptflops package 0.7.2.2 to calculate the multiply–accumulate operation (MAC).^15^ Parameters refer to the total count of weights, bias terms, and other parameters processed during the model’s training, with the unit of measurement being millions (M). A smaller number of parameters indicates a lower computational cost. MACs serve as a widely-used measure of computational complexity, especially in models that rely heavily on linear algebra operations like CNNs.^10^^,^^16^^,^^17^ In addition, we used the frames per second (fps) metric, an indicator used in previous studies,^16^ to represent the number of frames transmitted per second, which reflects the real-time nature of the ML model. Each model’s time required to process a single video clip was reported for comparative analysis at the video level. These additional metrics are crucial for understanding the feasibility of deploying the ML models in clinical environments where computational efficiency can be limited. Model training and inference were conducted on NVIDIA V100 graphic processing units.

### Statistical Analyses

We described the granularity characteristics at the video clip level using counts and proportions, such as the number of examinations and the number of patients with satisfactory and unsatisfactory videos in the training and independent datasets. Age was summarized as mean and standard deviation and gender as counts and proportions. It is important to note that a single examination may contain both satisfactory and unsatisfactory videos, so we did not perform comparative analyses at the video clip level. Normality tests was conducted on the age distribution in both datasets using the Shapiro-Wilk test. On the basis of the results of the normality tests, we used either the independent samples t-test or the Mann-Whitney U test to compare age differences between the 2 sets. For gender proportion differences, we employed the χ^2^ test for independence. In addition, we used Cohen’s κ coefficient^18^ to evaluate both intra-observer and inter-observer agreements on the annotations of the independent dataset between 2 cardiologists, and we created pairwise confusion matrices. All statistical analyses were performed using Python 3.1 and Sklearn 1.3.0.

## Result

### Dataset Composition and Quality Classification

The study’s characteristics are presented in Table 1. The training set consists of 1037 videos from 232 angiographic examinations involving 209 patients. The independent set comprises 20,388 videos from 3208 angiographic examinations involving 2725 patients. The distribution of data from 3 locations of Mayo Clinic is as follows: Minnesota (81.5%), Florida (10.7%), and Arizona (7.8%). There were no significant differences in the age and gender distributions between the 2 datasets, with the proportion of satisfactory videos being similar at 82.6% and 88.0%, respectively. In addition, the age and gender distributions of patients with satisfactory and unsatisfactory videos were also similar across both datasets (ages 62.7-65.4 years and genders 57.3%-64.6%). This similarity is attributed to the fact that most examinations included both satisfactory and unsatisfactory videos.

**Table 1:** Dataset Characteristics for Model Development and Independent Validation

	Train set (209 patients, 232 exams, and 1037 videos)		Independent Set (2725 patients, 3208 examinations, and 20,388 video)		*P*
	Satisfactory∗	Unsatisfactory∗	Satisfactory∗	Unsatisfactory∗	
Number of video clips, n (%)	857 (82.6)	180 (17.4)	17,937 (88.0)	2451 (12.0)	
Number of examinations, n (%)	150	104	3168	1180	
Number of patients, n (%)	135	96	2689	1118	
Age (y), mean ± SD	62.7±13.2	64.7±12.1	64.4±13.7	65.4±14.1	.18∗
Male gender, n (%)	77 (57.3)	58 (60.0)	1625 (60.4)	722 (64.6)	.49∗

The statistical comparisons were performed between the train set and the independent set.

∗ Satisfactory indicates the negative class and unsatisfactory indicates the positive class.

### Performance Metrics Across CNN Architectures

Table 2 summarizes the performance metrics of various models during development. In the ResNet-18 models, both 2D and 3D achieved an AUC of 0.97, whereas both the ResNet-152 2D and 3D models and the X3D-L model reached an AUC of 0.98. However, in other binary classification metrics, the 3D models generally outperformed the 2D models. Specifically, the ResNet 3D 18 and X3D-L models reported the best performance, with an accuracy of 0.96, precision of 0.90 and 0.87, and F1 scores of 0.93 and 0.92, respectively. In contrast, the 2D models found lower performance, with an accuracy ranging from 0.93 to 0.94, precision from 0.84 to 0.86, and F1 scores from 0.86 to 0.88. Table S1 presents the performance of the models at lower resolutions, with 2D using 299×299 and 3D using 160×160 image sizes. This exploratory model training aimed to evaluate how the architectures perform with smaller image resolutions. Most metrics for the ResNet 2D and 3D architectures found significant declines. The AUCs for 2D models were the lowest, at 0.89-0.93. Other metrics for ResNet 2D and 3D models, such as accuracy (0.85-0.88), precision (0.50-0.54), and F1 score (0.63-0.68), also decreased. The performance of the X3D-S architecture was similar to that of the X3D-L, with AUC and accuracy remaining consistent at 0.98 and 0.96, respectively, whereas precision and F1-score decreased to 0.86 and 0.87. Figure illustrates the adeptness of the X3D-CNN model in distinguishing between satisfactory and unsatisfactory angiograms within the test set.

**Table 2:** Comparative Performance Metrics of CNN Models

	AUC	Accuracy	Precision	Sensitivity	Specificity	F1-score
ResNet 2D 18	0.97	0.93	0.84	0.88	0.94	0.86
ResNet 2D 152	0.98	0.94	0.86	0.90	0.95	0.88
ResNet 3D 18	0.97	0.96	0.90	0.96	0.97	0.93
ResNet 3D 152	0.98	0.94	0.88	0.88	0.96	0.88
X3D-L	0.98	0.96	0.87	0.98	0.95	0.92

Abbreviations: AUC; area under the curve; CNN; convolutional neural network.

**Figure:**
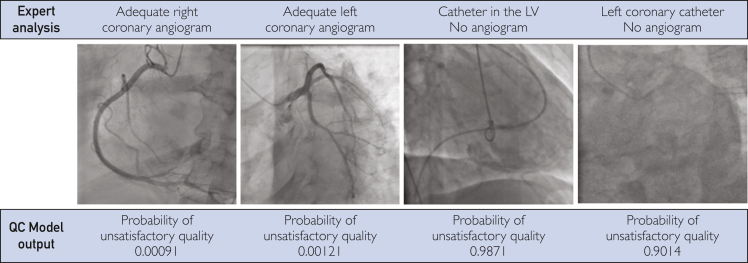
Examples of unsatisfactory versus satisfactory angiograms as assessed by cardiology experts with a corresponding output of the quality check 3D-CNN model. CNN, convolutional neural network; LV, left ventricle; QC, quality control.

### Computational Analysis of CNN Models

The parameter sizes of the 2D CNN models, ResNet-18 and ResNet-152, were 11.18 M and 58.15 M, with computational complexities of 9.53 Giga Multiply Accumulate Operation (GMAC) and 60.61 GMAC, respectively. During model inference, the ResNet-152 model processed videos significantly slower than the ResNet-18 model, with frame rates of 102.1 and 207.1 fps and processing times per video clip of 39.2 millisecond and 19.3 millisecond, respectively. For the 3D architectures, the parameter sizes of ResNet-18 and ResNet-152 increased substantially to 33.2 M and 117.41 M, and the computational complexities rose to 64.33 GMAC and 147.79 GMAC, respectively. These models processed data at rates of 233.9 fps and 206.1 fps, with each video clip taking 68.4 millisecond and 77.6 millisecond, respectively, reflecting the increased computational demands of 3D image analysis. Notably, the X3D-L model maintained a balance between computational efficiency and performance, with a parameter size of 5.34 M and a computational complexity of 19.34 GMAC. It achieved a processing speed of 235.1 fps and a processing time of 68.1 millisecond per video clip, highlighting its high performance with lower resource utilization than other 3D models (Table S2). When using a smaller image size, the computational complexity and the processing time per video clip both decreased. The processing times for the 2D models ranged from 19.32 to 24.31 millisecond, and for the 3D models, from 59.83 to 67.99 millisecond (Table S3).

### Validation of 3D CNNs on an Independent Dataset

We evaluated the performance of various models on the independent dataset (Table 3). The performance metrics indicate a relevant decline, particularly for the ResNet models, both 2D and 3D. Their AUC dropped to 0.80-0.93, accuracy to 0.83-0.88, precision to 0.39-0.50, and F1 score to 0.42-0.61. However, the X3D-L model maintained relatively better performance, with an AUC of 0.95, accuracy of 0.92, precision of 0.63, and F1 score of 0.71. The confusion matrix in Figure S1 shows that the X3D-L model correctly predicted 2004 unsatisfactory and 16,744 satisfactory angiograms and misclassifying 1193 satisfactory and 447 unsatisfactory cases, demonstrating reliable quality assessment with a low misclassification rate. Table S4 summarizes the performance of the 3D models trained on smaller image sizes on the independent dataset. The performance of these models was inferior to those trained on 312×312 images, with AUCs of 0.88-0.90 and F1 scores of 0.56-0.61.

**Table 3:** Predictive Outcomes on Independent Dataset

	AUC	Accuracy	Precision	Sensitivity	Specificity	F1-score
ResNet 2D 18	0.80	0.87	0.44	0.40	0.93	0.42
ResNet 2D 152	0.87	0.83	0.39	0.74	0.84	0.51
ResNet 3D 18	0.93	0.88	0.50	0.79	0.89	0.61
ResNet 3D 152	0.88	0.88	0.49	0.67	0.91	0.57
X3D-L	0.95	0.92	0.63	0.82	0.93	0.71

Abbreviation: AUC, area under the curve.

## Discussion

This research introduces an innovative and efficient deep learning approach for automating the selection of appropriate X-ray angiogram videos for ML-driven research in the Cath laboratory. It features a video selection method that benefits both the training of models and pre-inference screening. When evaluating various CNN models, 3D CNNs—particularly ResNet-18 and X3D-L—emerged as the top performers. Notably, the X3D-L model appears to strike an optimal balance between computational efficiency and complexity, rendering it highly suitable for clinical applications. This method was further validated using an independent dataset comprising 20,388 videos from 3208 angiographic studies, reporting the models’ reliability and practical applicability. This advancement offers an important contribution to cardiac imaging by enabling the automated selection of high-quality coronary angiogram videos, thereby enhancing the accuracy of artificial intelligence models in these fields.

There is a limited amount of literature regarding the auto-selection of suitable video X-ray images for training ML models. This could be attributed to the fact that past studies on angiograms mainly focused on delineating the coronary arteries, whereas training data has been commonly aligned with frame-by-frame labels. Nonetheless, when the model’s output pertains to cardiac function or predicting clinical events, it is often redundant to meticulously analyze and label each video clip of training data. Rather, each angiographic study is assigned a study-wise label for model training. This necessitates an automated method to help with excluding the inappropriate cine clips from each angiographic examination. A common approach involves setting rules on the basis of information in the Digital Imaging and Communications in Medicine headers, such as selecting the first few clips, ensuring the selection of a minimum number of frames, and specifying a particular frame rate. Other existing automated methods,^2^^,^^7^ such as keyframe selection and object detection suffer from major limitations that hinder their performance in selecting appropriate videos for input. Consequently, this study introduces an alternative and useful approach to automatically selecting qualified angiographic images for model inputs. This method may simplify the preparation of angiographic data for machine-learning applications, thereby facilitating more accurate and efficient model development.

In our study, evaluations were conducted on an independent dataset containing 20,388 video clips from 2725 patients, examining the both 2D and 3D architectures of ResNet-18 and ResNet-152, and X3D-L models. The X3D-L model reported the best performance. Taking the X3D-L model as an example, its automated screening recommended excluding 15.7% of videos, as opposed to a 12.0% exclusion rate seen with manual review. Among the videos classified as satisfactory by the model, 93.3% met the quality standards, indicating a high Negative Predictive Value (NPV). This NPV is significantly higher than the 88.0% observed when using all the data without selection. However, the application of the X3D-L model to the independent dataset led to a decrease in AUC from 0.98 to 0.95 and a reduction in precision from 0.87 to 0.63. Similar declines in performance were observed in the ResNet 2D and 3D models, highlighting the challenge of maintaining consistent performance across different datasets. Future applications of this selection model with downstream models on external datasets require rigorous evaluation of their combined generalizability in new environments.

The variability in model performance across datasets is considerably influenced by the diverse and complex nature of these datasets, complicating the establishment of a universal standard for classifying images as unsatisfactory. This complexity is further heightened in the case of coronary angiography images from patients with coronary artery bypass grafting, complicating the image selection process. Although graft angiograms from patients with previous operations were excluded, images where native vessels are clearly visible remained in consideration to preserve essential data. However, the post-operation appearance of native vessels can vary greatly, leading to potential exclusion without consistent criteria. Moreover, the task of accurately delineating coronary arteries is complicated by the presence of foreign objects in the image, which can obscure crucial details. This adds another layer of complexity to maintaining uniform evaluation standards across different datasets. Despite these challenges, our annotations maintained high agreement levels, with intra-observer and inter-observer Cohen’s κ coefficients ranging from 0.80 to 0.87 and 0.81 to 0.87, respectively (Table S5, Figure S2). We recommend using the same automated image selection model during inference to preprocess input images to ensure optimal inference accuracy. This approach will help maintain input quality consistency and maximize the downstream models’ predictive performance.

In the same ResNet architecture, although the 2D models have a smaller structure and lower computational complexity compared with the 3D models, they exhibit lower fps. This may be attributed to the inherent differences in processing spatiotemporal data: 2D models handle each frame independently and merge the results, which may lead to inefficiencies, whereas 3D models process multiple frames in sequence, directly yielding predictions. Although 3D models have higher computational demands, they can benefit from optimized graphic processing unit acceleration and improved architectural designs, as seen in the X3D models. Despite this, the 3D architecture processes more frames, resulting in longer processing times for each video clip than their 2D counterparts. Notably, although X3D is a 3D CNN, it has fewer parameters than the ResNet 2D models, with computational complexity between ResNet 2D 18 and ResNet 2D 152, and it is the fastest among the 3D architectures in terms of processing speed.

When the image size is reduced, the models show improved fps and processing time per video. For example, the fps and processing time of the X3D architecture improved by ∼12%, but the prediction accuracy decreased significantly. In practical applications, we typically perform model inference on 10 video clips from a coronary angiographic examination. Using X3D-L, this process takes 0.68 seconds, whereas using X3D-S only reduces the time by 0.08 seconds. Therefore, choosing X3D-L represents a more balanced option.

### Limitations

This study’s limitations stem from using datasets exclusively from the Mayo Clinic limiting the model’s generalizability without external validation. Despite this, the model performed well across 3 Mayo Clinic’s locations. Furthermore, from the perspective of developing models using satisfactory angiographic videos, a small subset of videos could be selected to train this quality selection tool. This tool can then be applied to the rest part of the entire large dataset for quality screening, subsequently providing high-quality videos for further model development. In this scenario, this quality screening model does not require cross-institutional generalizability. However, our study did not perform a direct comparison between ML models trained on manually curated datasets and those trained on the automatically selected datasets, which should be explored in future research to further validate the method's applicability. In future studies, fostering inter-institutional collaborations to gather cardiac catheterization X-ray images from varied regions and populations could significantly broaden the model's applicability. Moreover, leveraging transfer learning^19^ strategy or using attention-based mechanisms^20^ may improve the model's adaptability and precision effectively.

## Conclusion

This research presents a streamlined and efficient methodology for creating an automated 3D CNN-based quality evaluation tool for video coronary angiograms, exhibiting relevant accuracy and effectiveness. The principal advantage of this model is its facilitation of accelerated ML model development within Cath laboratory research domains, particularly for studies necessitating extensive video angiogram analysis. Furthermore, it possesses the potential to automate input selection for models in real-world applications, thereby augmenting predictive precision.

## Potential Competing Interest

The authors report no competing interests.
