# Supplementary material for: Optimizing Input Selection for Cardiac Model Training and Inference: An Efficient 3D Convolutional Neural Networks-Based Approach to Automate Coronary Angiogram Video Selection
Source: Mayo Clin Proc Digit Health. 2025 Jan 21;3(1):100195. doi: 10.1016/j.mcpdig.2025.100195 (PMC11975815; doi:10.1016/j.mcpdig.2025.100195)
Supplement: Supplemental Material [file mmc1.pdf]

Supplemental Material

Model Training

To avoid data leakage, we organized the angiogram images into training, validation, and test sets using unique Patient IDs, allocating 70% for training, and 15% each for validation and testing. This study utilized both 2D and 3D CNN architectures (ResNet-18, ResNet-152, and X3D) to assess the quality of the angiograms. These models were chosen due to their proven capabilities in processing visual information and extracting image features, which are particularly beneficial for video analysis.

DICOM images were converted to PNG format and resized to ensure compatibility with the model. For the primary 3D CNN model, images were resized to 312x312 pixels, with exploratory experiments using images resized to 160x160 pixels. For the primary 2D CNN model, images were resized to 512x512 pixels, and exploratory experiments utilized images resized to 299x299 pixels. We selected videos with a frame rate of 15 frames per second based on DICOM header information, ensuring consistent temporal resolution for analysis. To capture the most representative frames from angiogram videos, we selected 32 frames starting from the 16th frame of each video clip. This range was chosen to avoid initial frames which often lack relevant content and final frames which can be affected with contrast washout. For the 2D CNN-based experiments, one frame per eight frames within this range was selected, providing comprehensive coverage of the video content. In contrast, with 3D-CNNs, we sampled every other frame, considering the minimal variation between consecutive frames and focusing on image quality assessment rather than detailed temporal relationships.

These models were trained using Python 3.10.12 and the PyTorch framework version 1.12.1, with the training process spanning 100-200 epochs. We employed the Adam optimizer along with a step decay learning rate schedule (StepLR) with a step size of 30 epochs. The learning rate for all models was set between 1e-6 and 1e-4, with a weight decay factor of 1e-3 and a batch size ranging from 8 to 64. Due to GPU constraints, the batch size had to be reduced when using larger image sizes. Additionally, we did not utilize image sizes larger than 312x312 pixels for the 3D CNN training due to GPU limitations. For the X3D models, the X3D-S and X3D-L architectures used image sizes of 160x160 and 312x312 pixels, respectively. To further optimize the models, we applied data augmentation techniques, including flipping, rotating, perspective changes, Gaussian blurring, and image normalization. These methods were implemented to simulate real clinical variations and enhance the models' ability to adapt to actual clinical scenarios.

Table S1 Comparative Performance Metrics of CNN Models Trained with Reduced Image Sizes

	AUC	Accuracy	Precision	Sensitivity	Specificity	F1-score
**ResNet 2D 18**						
*299x299**	0.89	0.85	0.5	0.86	0.85	0.63
**ResNet 2D 152**						
*299x299**	0.93	0.88	0.57	0.81	0.89	0.67
**ResNet 3D 18**						
*160x160**	0.95	0.87	0.51	**0.95**	0.85	0.67
**ResNet 3D 152**						
*160x160**	0.97	0.88	0.54	0.9	0.88	0.68
**X3D-S**						
*160x160**	**0.98**	**0.96**	**0.86**	0.89	**0.98**	**0.87**

* Images were preprocessed and resized to the specified dimensions.

Abbreviations: CNN; convolutional neural network, AUC; area under the curve.

Table S2. Model Complexity and Computational Efficiency

	Parameters (M)	MAC (G)	FPS	Processing Time Per Clip (ms)
**ResNet 2D 18**	11.18	**9.53**	207.1	**19.3**
**ResNet 2D 152**	58.15	60.61	102.1	39.2
**ResNet 3D 18**	33.2	64.33	233.9	68.4
**ResNet 3D 152**	117.41	147.79	206.1	77.6
**X3D-L**	**5.34**	19.34	**235.1**	68.1

Abbreviations: M, million; FLOPs, floating-point operations per second; MAC, multiply-accumulate operations; G, giga; FPS, frames per second; ms, millisecond.

Table S3 Model Complexity and Computational Efficiency of CNN Models Trained with Reduced Image Sizes

	**Parameters (M)**	**MAC (G)**	**FPS**	**Processing Time Per Clip (ms)**
**ResNet 18 2D**				
*299x299**	11.18	3.41	164.5	24.31
**ResNet 152 2D**				
*299x299**	58.15	21.46	207.1	**19.32**
**ResNet18 3D**				
*160x160**	33.2	59.79	**268.8**	67.99
**ResNet 152 3D**				
*160x160**	117.41	136.11	239.2	66.90
**X3D-S**				
*160x160**	**2.98**	**2.56**	267.4	59.83

* Images were preprocessed and resized to the specified dimensions.

Abbreviations: M, million; FLOPs, floating-point operations per second; MAC, multiply-accumulate operations; G, giga; FPS, frames per second; ms, millisecond.

Table S4 Predictive Outcomes of 3D CNN Models Trained with Reduced Image Sizes on Independent Dataset

	AUC	Accuracy	Precision	Sensitivity	Specificity	F1-score
**ResNet 3D 18** *160x160**	**0.90**	0.87	0.48	**0.78**	0.88	0.59
**ResNet 3D 152** *160x160**	0.88	0.86	0.44	0.74	0.87	0.56
**X3D-S** *160x160**	**0.90**	**0.88**	**0.49**	**0.78**	**0.89**	**0.61**

* Images were preprocessed and resized to the specified dimensions.

Abbreviations: AUC, area under the curve.

Table S5 Intra-Observer and Inter-Observer Agreements for Different Annotators on Independent Dataset

Agreement Types	Annotator-1's Subset	Annotator-2's Subset
**Intra-observer (Cohen's Kappa)**	0.80	0.87
**Inter-observer (Cohen's Kappa)**		
Scenario 1[a]	0.81	0.85
Scenario 2[b]	0.81	0.87

a The non-original annotator's labeling results compared to the original annotator's labeling results.

b The non-original annotator's labeling results compared to the original annotator's second labeling results.

Figure S1 Confusion Matrices for Intra-Observer and Inter-Observer Agreements of Different Annotators on Independent Dataset

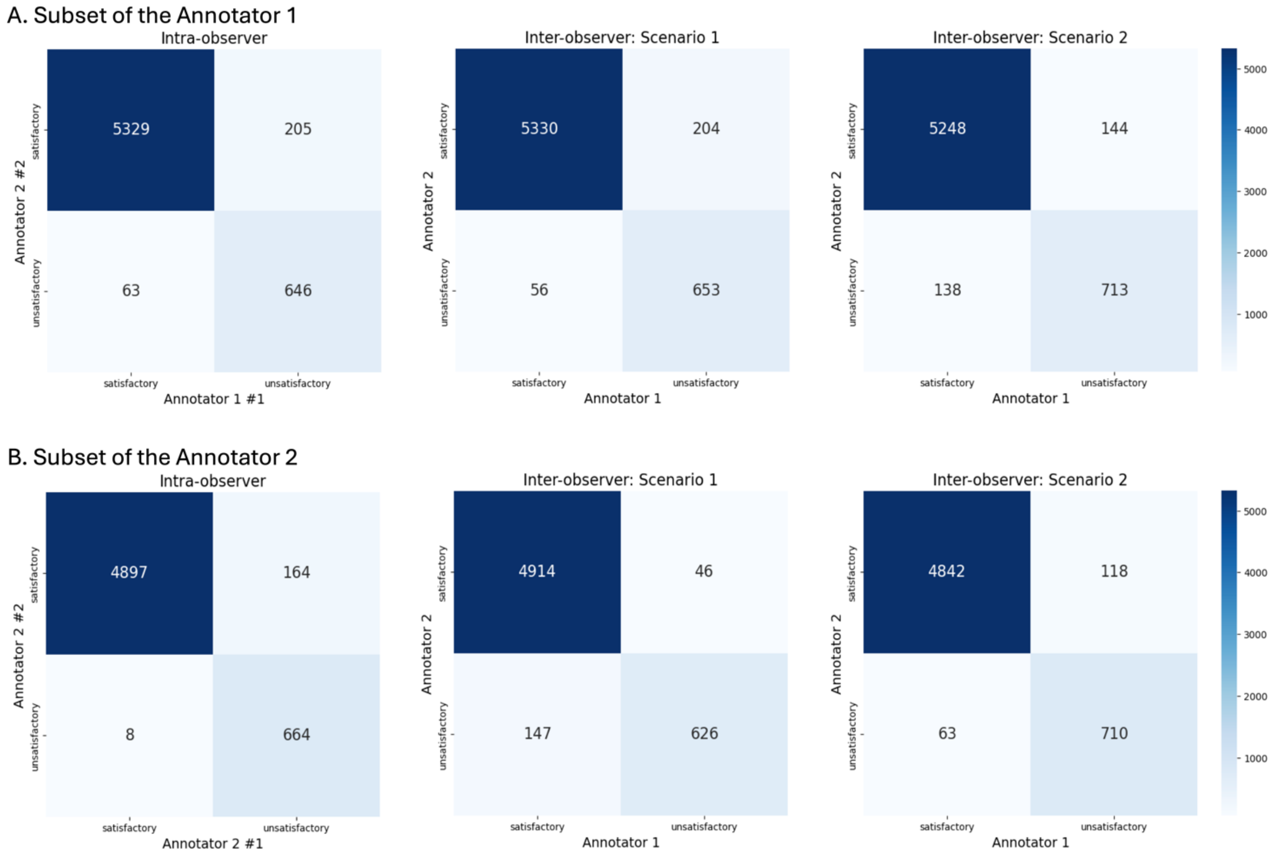

Subset of Annotator 1 (A) and Subset of Annotator 2 (B) represent the subsets originally labeled by Annotator 1 and Annotator 2, respectively. #1 and #2 indicate the first and second labeling sessions by the same annotator on their respective subsets. Scenario 1 indicates the comparison between the non-original annotator's labeling results and the original annotator's first labeling results. Scenario 2 indicates the comparison between the non-original annotator's labeling results and the original annotator's second labeling results.

Figure S2 Confusion Matrix of The X3D-L Model Applying The Independent Datase

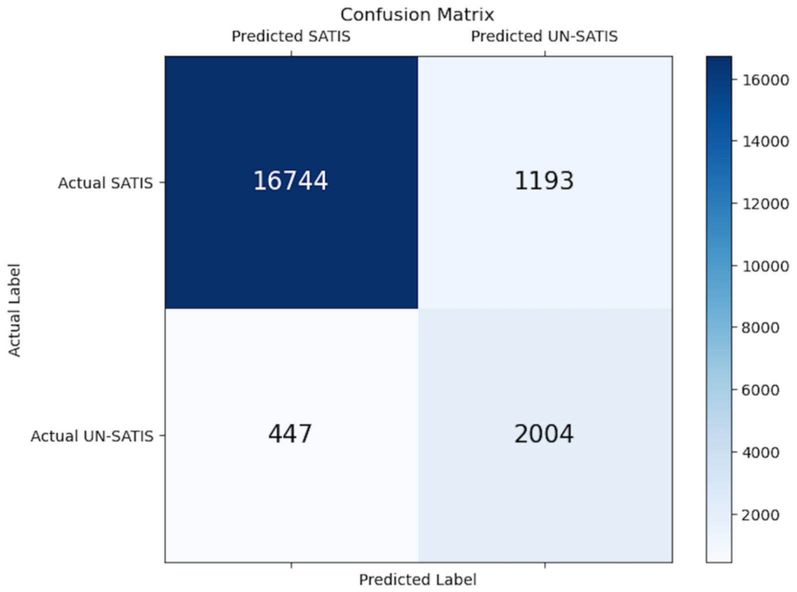

Abbreviation: SATIS, satisfactory; UN-SATIS, unsatisfactory.
